# Variation in treatment and survival of older patients with non-metastatic breast cancer in five European countries: a population-based cohort study from the EURECCA Breast Cancer Group

**DOI:** 10.1038/s41416-018-0090-1

**Published:** 2018-06-07

**Authors:** Marloes G. M. Derks, Esther Bastiaannet, Mandy Kiderlen, Denise E. Hilling, Petra G. Boelens, Paul M. Walsh, Elizabeth van Eycken, Sabine Siesling, John Broggio, Lynda Wyld, Maciej Trojanowski, Agnieszka Kolacinska, Justyna Chalubinska-Fendler, Ana Filipa Gonçalves, Tomasz Nowikiewicz, Wojciech Zegarski, Riccardo A. Audisio, Gerrit-Jan Liefers, Johanneke E. A. Portielje, Cornelis J. H. van de Velde, on behalf of the EURECCA Breast Cancer Group

**Affiliations:** 1 Department of Surgery, Leiden University Medical Center, Albinusdreef 2, Leiden, 2333 ZA The Netherlands; 2 Department of Gerontology & Geriatrics, Leiden University Medical Center, Albinusdreef 2, Leiden, 2333 ZA The Netherlands; 3 National Cancer Registry of Ireland, Building 6800, Cork Airport Business Park, Kinsale Road, Cork, T12 CDF7 Ireland; 4 Belgian Cancer Registry, Koningsstraat 215, Brussels, 1210 Belgium; 5 Department of Research, Netherlands Comprehensive Cancer Organisation (IKNL), Godebaldkwartier 419, Utrecht, 3511 DT The Netherlands; 6 Public Health England, 5 St Philip’s Place, Birmingham, B3 2PW UK; 7 Department of Oncology and Metabolism, University of Sheffield, Western Bank, Sheffield, S10 2TN UK; 8 Greater Poland Cancer Registry, Greater Poland Cancer Center, Garbary 15, Poznań, 60-101 Poland; 9 Department of Head and Neck Cancer Surgery, Department of Surgical Oncology, Medical University of Lodz, Kościuszki 4, Łódź, 90-419 Poland; 10 Department of Radiotherapy, Medical University of Lodz, Kościuszki 4, Łódź, 90-419 Poland; 11 Portuguese Oncology Institute of Porto, R. Dr. António Bernardino de Almeida 62, Porto, 4200-162 Portugal; 12 Department of Surgical Oncology, Ludwik Rydygier’s Collegium Medicum, Jagiellońska 13-15, Bydgoszcz, 85-067 Poland; 13 Department of Surgery, St Helens Teaching Hospital, University of Liverpool, Marshalls Cross Rd, Saint Helens, St Helens, WA9 3DA UK; 14 Department of Medical Oncology, Leiden University Medical Center, Albinusdreef 2, Leiden, 2333 ZA The Netherlands

**Keywords:** Breast cancer, Cancer epidemiology, Breast cancer

## Abstract

**Background:**

Older patients are poorly represented in breast cancer research and guidelines do not provide evidence based recommendations for this specific group. We compared treatment strategies and survival outcomes between European countries and assessed whether variance in treatment patterns may be associated with variation in survival.

**Methods:**

Population-based study including patients aged ≥ 70 with non-metastatic BC from cancer registries from the Netherlands, Belgium, Ireland, England and Greater Poland. Proportions of local and systemic treatments, five-year relative survival and relative excess risks (RER) between countries were calculated.

**Results:**

In total, 236,015 patients were included. The proportion of stage I BC receiving endocrine therapy ranged from 19.6% (Netherlands) to 84.6% (Belgium). The proportion of stage III BC receiving no breast surgery varied between 22.0% (Belgium) and 50.8% (Ireland). For stage I BC, relative survival was lower in England compared with Belgium (RER 2.96, 95%CI 1.30–6.72, *P* < .001). For stage III BC, England, Ireland and Greater Poland showed significantly worse relative survival compared with Belgium.

**Conclusions:**

There is substantial variation in treatment strategies and survival outcomes in elderly with BC in Europe. For early-stage BC, we observed large variation in endocrine therapy but no variation in relative survival, suggesting potential overtreatment. For advanced BC, we observed higher survival in countries with lower proportions of omission of surgery, suggesting potential undertreatment.

## Introduction

Cancer is a disease of the elderly; 30% of patients diagnosed with breast cancer (BC) are aged 70 years or older^1^. Although this group of older patients is rapidly growing, evidence to guide treatment of these patients remains scarce^2^. Clinical trials often have inclusion criteria that preclude older patients from participating^3^. Furthermore, older patients participating in trials may not be representative for the wider older population owing to selection of fitter older patients, those with higher socio-economic status and those with good cognitive function. These differences impair the external validity of trials and limit the extrapolation of their findings^4^.

The American Society of Clinical Oncology (ASCO) and the International Society of Geriatric Oncology (SIOG) have called for age specific clinical trials to improve treatment in this patient group^3,5^. However, de Glas and colleagues^6^ showed that only 4% of the currently running trials for BC treatment are specifically including older patients. Therefore, major improvement in the evidence base for treatment in older patients is not likely to occur within a short period of time. An alternative way to study treatment in older patients is by using observational data. Observational data from cancer registries are highly representative for the older population because there is no selection for inclusion^4^. Furthermore, observational data are currently available and can directly be used for research purposes^7^. They provide better insight into treatment strategies and, when using appropriate methods, may be used to evaluate the efficacy of different treatment strategies^8^. For these reasons, the European Registration of Cancer Care project (EURECCA) Breast Cancer Group, collected data from cancer registries on treatment and survival outcomes in older patients with BC.

The aim of this study was to compare differences in locoregional and systemic treatment patterns and survival outcomes in older patients with non-metastatic BC across five European countries. In addition, this study aimed to assess whether variance in treatment between countries was associated with outcome variation.

## Materials and Methods

This is an observational cohort study with data obtained from four national (The Netherlands, Belgium, Ireland and England) and one regional (Greater Poland) population-based cancer registry. All patients aged 70 years and older at time of diagnosis with non-metastatic invasive BC were selected. The International Classification of Diseases and Related Health Problems (ICD-10) coding was used for selection of BC^9^. In case of synchronous or bilateral tumours, the tumour with the highest known TNM stage was selected for analysis. In addition, second primary tumours and patients diagnosed with BC only at the time of death were excluded.

### Procedures

The protocol specified that data on all consecutive BC cases available between 2000 and 2013 should be provided with information on stage of disease, treatment and vital status. For all national and regional based CRs coverage rate was ~ 100%. Quality of the CRs and methods and periods of collection of the data are described in Supplementary Table S1.

Stage of disease was defined using the TNM Classification of Malignant Tumours for BC, 6th edition^10^. Information on tumour stage was based on pathology reports. If the pathological T or N category was unknown, clinical stage was used instead. For patients with unknown T or N category (both clinical and pathological) stage of disease was considered unknown, unless patients with only known T or N category could be reliably assigned to a specific stage (for example, T4NXMX = stage III). Patients with an unknown M-category were assumed to have non-metastatic disease (unless T and N category were both unknown). When stage directly derived from patient reports was available but was assigned unknown according to the above mentioned stage definition, stage available from reports was used instead. If available, data on tumour grade, morphology and hormone receptor expression were collected. Tumour grade was classified as grade I (well differentiated), grade II (moderately differentiated) or grade III (poorly differentiated). Morphology was classified into ductal, lobular, or mixed/other according to ICD-O-3 classification^11^.

### Outcomes

Main outcomes were the proportion of given treatment for locoregional treatment (breast surgery, axillary surgery and radiotherapy) and systemic treatment (endocrine therapy, chemotherapy and primary endocrine therapy) and 5-year relative survival for each country. Breast surgery was defined as the most extensive breast surgery (no surgery, breast-conserving surgery (BCS), mastectomy, breast surgery not otherwise specified), axillary surgery if any breast surgery (yes or no) and radiotherapy if BCS (yes or no). Adjuvant endocrine therapy was defined as endocrine therapy if any breast surgery was performed (yes or no). Adjuvant chemotherapy was defined as chemotherapy if any breast surgery was performed (yes or no). Most registries did not distinguish between adjuvant or neo-adjuvant systemic therapy. Therefore, these were combined. Primary endocrine therapy was defined as endocrine therapy without receiving surgery (yes or no). Vital status was provided by the CRs and defined as alive, dead, or unknown. Follow-up time for vital status was defined as time in days from diagnosis until death or end of follow-up. Vital status and date of last follow-up were established either directly from the patient’s medical record or through linkage of cancer registry data with mortality or population registries (Supplementary Table S1). All outcomes were stratified for stage (I–III).

### Statistical analysis

All analyses were performed in Stata/MP. Data from national or regional data registries were compared between countries. Proportions of patients undergoing each treatment were calculated. Owing to the large number of cases, no statistical tests were conducted to assess statistical significant proportional differences. Median follow-up and interquartile range (IQR) were calculated according to the reverse Kaplan–Meier method^12^. Relative survival reflects the ratio of overall survival of cancer patients compared with survival that would have been expected based on the corresponding general population (matched by country, age by single year and year of diagnosis). Relative survival for the complete cohort was estimated using the Pohar-Perme method^13^. National life tables from The Human Mortality Database were used to estimate expected survival^14^. To model the effect of covariates on relative survival an additive hazard model was employed. The effect of covariates on the excess hazard was estimated using the expectation-maximisation method^15^. Estimates of the covariates are expressed as relative excess risk of death (RER) and they quantify the relative cancer related excess mortality between the categories of the included covariates in the model^16^. When the excess mortality is low (for instance in a population with a high population mortality and generally curable cancer), standard errors become large and hamper the interpretation of the RER^15^. To compare RER between countries, country was included as a covariate in the univariate model. Differences in relative survival between countries were adjusted for the following potential confounders in a multivariable model: age (continuous), year of diagnosis, stage (not when stratified for stage), grade and morphology. A two-sided *p* value of < 0.05 was considered statistically significant. In Table 3 and Fig. 2, countries were ranked according to the sum of proportions of given treatment and the country with the highest sum was assigned as reference country.

**Fig. 1:**
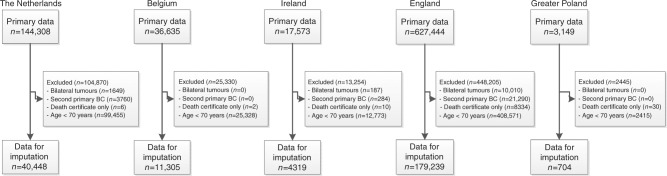
Flow chart. Bilateral tumours: in case of synchronous tumours, the smallest stage tumour was excluded

Multiple imputation was used to account for missing values for each country separately after exclusion of tumours diagnosed at time of death, second primary BC and smaller synchronous tumours and age younger than 70 years (Fig. 1). Multiple imputation by chained equation was performed, assuming that data are missing at random. For each incomplete variable (stage, grade, morphology, hormone receptor expression), imputation models were applied that included the other incomplete variables, as well was complete variables (age, year of diagnosis), treatment variables and outcome variables (vital status, follow-up time in days). When data for a variable was 100% missing it was not imputed. Analyses were based on pooled results of five imputed data sets^17^.

### Additional analyses

A sensitivity analysis was performed to assess the impact of variation in time periods on treatment and survival outcomes between the participating countries only including the years with data available from all countries (2008 and 2009). Based on expert panel discussion, a proportional difference of 10% or higher between treatment outcomes was defined as clinically relevant.

### Ethical approval

Data from cancer registries provided anonymised patient data. Therefore, informed consent from patients or ethical approval were not required for this study.

## Results

### Patients

The original data set included 829,131 patients diagnosed with BC between 2000 and 2013. Patients with synchronous or bilateral tumours, second primary tumours, tumours diagnosed at time of death and patients aged younger than 70 years were excluded (Fig. 1). A total of 40,448 patients from the Netherlands, 11,305 patients from Belgium, 4319 patients from Ireland, 179,239 patients from England and 704 patients from Greater Poland were included (Table 1, step 1). Multiple imputation analysis was performed to account for missing values (Table 1, step 2) and selected patients stage I–III BC for further analyses (Table 1, step 3). Median follow-up was 8.8 years (IQR 5.9–12.5 years).

**Table 1:** Distribution of patient and tumour characteristics by country, before and after imputation (Step 1 and 2) and after selection of patients with stage I–III breast cancer (Step 3)

	Netherlands				Belgium				Ireland				England				Greater Poland			
	Step 1		Step 2	Step 3	Step 1		Step 2	Step 3	Step 1		Step 2	Step 3	Step 1		Step 2	Step 3	Step 1		Step 2	Step 3
	*N*	%	%	%	*N*	%	%	%	*N*	%	%	%	*N*	%	%	%	*N*	%	%	%
Total *N*	40,448				11,305				4319				179,239				704			
Year of diagnosis																				
2000	3745	9.3	9.3	9.2	0	0.0	0.0	0.0	0	0.0	0.0	0.0	11837	6.6	6.6	6.4	0	0.0	0.0	0.0
2001	3688	9.1	9.1	9.1	0	0.0	0.0	0.0	0	0.0	0.0	0.0	12073	6.7	6.7	6.6	0	0.0	0.0	0.0
2002	3555	8.8	8.8	8.7	0	0.0	0.0	0.0	0	0.0	0.0	0.0	11995	6.7	6.7	6.7	0	0.0	0.0	0.0
2003	3553	8.8	8.8	8.7	0	0.0	0.0	0.0	533	12.3	12.3	12.4	12409	6.9	6.9	6.9	0	0.0	0.0	0.0
2004	3656	9.0	9.0	9.0	0	0.0	0.0	0.0	566	13.1	13.1	13.1	12302	6.9	6.9	6.8	0	0.0	0.0	0.0
2005	3609	8.9	8.9	8.9	0	0.0	0.0	0.0	567	13.1	13.1	12.7	12935	7.2	7.2	7.1	0	0.0	0.0	0.0
2006	3590	8.9	8.9	8.9	0	0.0	0.0	0.0	638	14.8	14.8	14.6	12666	7.1	7.1	7.0	0	0.0	0.0	0.0
2007	3771	9.3	9.3	9.5	2763	24.4	24.4	24.7	641	14.8	14.8	15.0	12645	7.1	7.1	7.0	0	0.0	0.0	0.0
2008	3797	9.4	9.4	9.4	2805	24.8	24.8	24.7	662	15.3	15.3	15.2	12994	7.2	7.2	7.2	325	46.2	46.2	47.9
2009	3666	9.1	9.1	9.1	2842	25.1	25.1	24.9	712	16.5	16.5	17.0	12902	7.2	7.2	7.2	379	53.8	53.8	52.1
2010	3818	9.4	9.4	9.5	2895	25.6	25.6	25.7	0	0.0	0.0	0.0	13231	7.4	7.4	7.4	0	0.0	0.0	0.0
2011	0	0.0	0.0	0.0	0	0.0	0.0	0.0	0	0.0	0.0	0.0	13294	7.4	7.4	7.5	0	0.0	0.0	0.0
2012	0	0.0	0.0	0.0	0	0.0	0.0	0.0	0	0.0	0.0	0.0	13685	7.6	7.6	7.9	0	0.0	0.0	0.0
2013	0	0.0	0.0	0.0	0	0.0	0.0	0.0	0	0.0	0.0	0.0	14271	8.0	8.0	8.2	0	0.0	0.0	0.0
Stage																				
0	5	0.0	0.4		11	0.1	0.1		0	0.0	0.0		7727	4.3	8.8		19	2.7	9.9	
I	14416	35.6	36.1	39.0	2986	26.4	28.7	32.4	740	17.1	21.3	25.6	29581	16.5	26.6	33.7	113	16.1	19.6	28.7
II	17234	42.6	43.2	46.6	4333	38.3	42.3	47.8	1553	36.0	42.3	50.8	44115	24.6	39.2	49.7	190	27.0	31.4	45.9
III	5159	12.8	13.3	14.4	1779	15.7	17.5	19.8	664	15.4	19.7	23.6	13091	7.3	13.1	16.6	110	15.6	17.3	25.3
IV	2662	6.6	7.0		918	8.1	11.4		444	10.3	16.8		8200	4.6	12.3		110	15.6	21.8	
Unknown	972	2.4			1278	11.3			918	21.3			76525	42.7			162	23.0		
Grade																				
G1	6839	16.9	22.1	22.9	1399	12.4	15.0	15.6	370	8.6	10.3	11.1	21261	11.9	16.5	16.8	82	11.6	20.1	23.5
G2	14376	35.5	48.2	48.9	4414	39.0	48.0	48.1	2102	48.7	57.9	57.6	73916	41.2	53.5	54.5	176	25.0	48.3	46.7
G3	8245	20.4	28.5	28.2	3549	31.4	37.0	36.3	1169	27.1	31.7	31.3	42223	23.6	30.0	28.7	135	19.2	31.6	29.8
Unknown	10988	27.2			1943	17.2			678	15.7			41839	23.3			311	44.2		
Morphology																				
Ductal	25812	63.8	63.8	65.2	8058	71.3	71.3	71.6	2771	64.2	64.2	65.3	115345	64.4	64.4	66.7	401	57.0	59.6	69.3
Lobular	5276	13.0	13.0	12.9	1643	14.5	14.5	14.3	591	13.7	13.7	13.9	21634	12.1	12.1	12.8	53	7.5	7.7	8.8
Mixed/other	9360	23.1	23.1	21.9	1604	14.2	14.2	14.1	957	22.2	22.2	20.8	42260	23.6	23.6	20.5	189	26.8	32.7	21.9
Unknown	0	0.0			0	0.0			0	0.0			0	0.0			61	8.7		
Hormone receptor expression																				
ER− and PR−	2798	6.9	14.4	14.1	0	0.0	0.0	0.0	570	13.2	16.6	16.4	6823	3.8	16.1	15.1	115	16.3	28.5	23.8
ER + and/or PR+	18576	45.9	85.6	85.9	0	0.0	0.0	0.0	3142	72.7	83.4	83.6	44586	24.9	83.9	84.9	380	54.0	71.5	76.2
Unknown	19074	47.2			11305	100.0	100.0	100.0	607	14.1			127830	71.3			209	29.7		

Step 1: Distribution of patients aged 70 years and older by category before imputation; Step 2: Distribution of patients aged 70 years and older by category after imputation; Step 3: Distribution of patients aged 70 years and older with stage I-III breast cancer by category after imputation

### Patient characteristics

Stage distribution varied slightly across countries; patients from the Netherlands were more frequently diagnosed with stage I BC compared with other countries (Table 1, step 3). Overall, tumour characteristics were broadly comparable across countries (Table 1, step 3). Patients from the Netherlands and Greater Poland were more likely to have grade I BC.

### Locoregional treatment

**Fig. 2:**
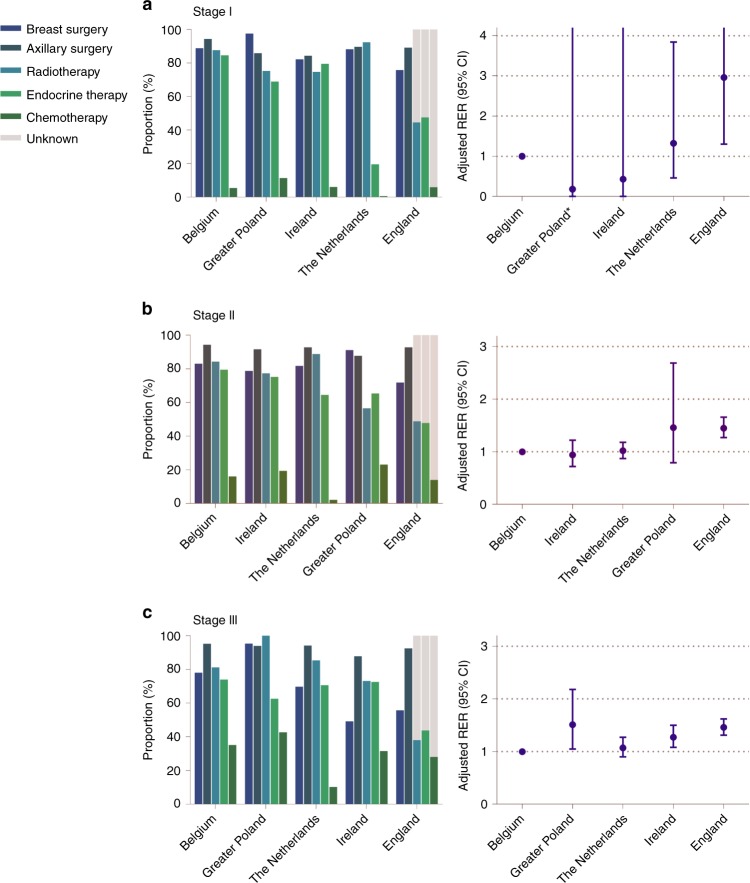
Proportion of patients receiving treatment and adjusted relative excess risks (RERs) of death by stage of disease. Proportions of patients receiving therapy and adjusted relative excess risks (RER) of death by country for patients with stage I **a**, stage II **b**, or stage III **c** breast cancer. Countries were ranked according to the sum of proportions of each given treatment and the country with the highest sum of given treatment was assigned as reference country. Breast surgery: % of patients receiving any type of breast surgery; axillary surgery: % of patients receiving axillary surgery if they received any type of breast surgery; radiotherapy: % of patients receiving radiotherapy if they have received breast-conserving surgery; endocrine therapy: % of patients receiving endocrine therapy if they have received any type of breast surgery; chemotherapy: % of patients receiving chemotherapy if they have received any type of breast surgery. Error bars represent 95% confidence intervals. RER was adjusted for the following variables: age, year of diagnosis, grade, and morphology

As shown in Table 2, the majority of patients with stage I BC received BCS (between 48.9% (England) and 65.1% (Belgium), except for Greater Poland (21.1%)). Omission of surgery was commonly used in England (24.2%) and Ireland (17.8%) compared with other countries. For stage II BC, the majority of patients received a mastectomy (between 44.0% (Ireland) and 66.1% (Greater Poland)). The proportions of patients not receiving any surgery showed a similar pattern as seen in patients with stage I BC (Table 2). For stage III BC, the proportion of patients not receiving any surgery increased compared with lower stages of BC: this is most pronounced in The Netherlands (30.1%), England (44.1%) and Ireland (50.8%). The majority of patients who had breast surgery received axillary treatment with no clinically relevant differences between countries and across stages (Fig. 2, Supplementary Table S2). In England (across all stages) and Greater Poland (for stage III), the proportion of patients receiving radiotherapy after BCS was lower (Fig. 2, Supplementary Table S2).

**Table 2:** Proportional distribution of most extensive breast surgery by stage of disease

	No surgery	BCS	Mastectomy	Not specified
	%	%	%	%
Stage I				
The Netherlands	11.7	50.3	38.0	0.0
Belgium	11.1	65.1	23.8	0.0
Ireland	17.8	54.4	27.8	0.0
England	24.2	48.9	26.9	0.0
Greater Poland	2.5	21.1	52.4	24.0
Stage II				
The Netherlands	18.2	22.3	59.5	0.0
Belgium	16.9	35.8	47.3	0.0
Ireland	21.2	34.8	44.0	0.0
England	28.1	27.5	44.4	.0
Greater Poland	8.9	8.3	66.1	16.7
Stage III				
The Netherlands	30.1	8.3	61.5	0.0
Belgium	22.0	14.4	63.6	0.0
Ireland	50.8	10.4	38.8	0.0
England	44.1	9.5	46.3	0.0
Greater Poland	4.6	3.4	81.8	10.2

### Systemic treatment

**Fig. 3:**
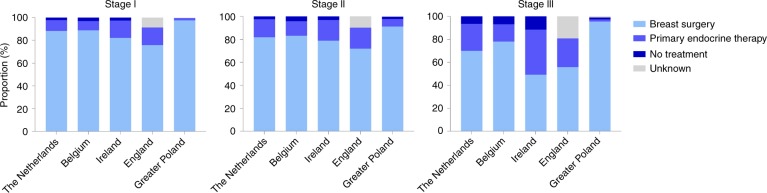
Proportion of patients receiving breast surgery, primary endocrine therapy or no therapy by stage of disease

Use of adjuvant endocrine therapy differed considerably between countries: for stage I BC the proportion was substantially lower in the Netherlands (20%), compared with the other countries (Belgium 84.6%; Ireland 79.5%; England 47.5%; Greater Poland 68.9%, Fig. 2a, Supplementary Table S2). In England, systemic therapy was not registered for a large proportion of patients but this could not be considered as not given, hence this is considered as unknown (Fig. 2). For higher stages of BC, variation was less pronounced between countries (Figs. 2b,C, Supplementary Table S2). In addition, substantial variation in the administration of chemotherapy across countries was observed. The proportion of patients with stage I BC receiving chemotherapy was very low across all countries but showed marked variation (range from 0.5% (the Netherlands) to 6.0% (Ireland) and 11.4% (Greater Poland), Fig. 2a, Supplementary Table S2). For stage II BC, chemotherapy use was higher but again varied markedly between countries (range from 2.2% (the Netherlands) to 19.4% (Ireland) and 23.1% (Greater Poland), Fig. 2b, Supplementary Table S2). For stage III BC, chemotherapy use increased further but still varied markedly, from 10.3% of patients in the Netherlands to 35.2% in Belgium and 42.7% in Greater Poland (Fig. 2c, Supplementary Table S2). As shown in Fig. 2, use of primary endocrine therapy (PET) was a commonly used strategy among older patients with BC (Fig. 3, Supplementary Table S3). In stage III, disease differences between countries were most pronounced; in Ireland 39% of the patients received primary endocrine therapy, compared with 23.6% in the Netherlands, 24.9% in England, 15.1% in Belgium and 1.8% in Greater Poland (Fig. 3, Supplementary Table S3).

### Survival outcomes

As shown in Table 3, 5-year relative survival for patients with stage I BC was high for all countries, indicating that there is little to no excess mortality in this stage of disease. For England, relative survival was significantly lower compared with Belgium (93.4% 95% CI 93.1–93.7, adjusted RER 2.96, *P* < 0.001). Owing to low excess mortality in this specific group, RERs for some countries could not be estimated (Table 3, Fig. 2a). For patients with stage II BC, 5-year relative survival was lowest in England (79.1%, 95% CI 78.8–79.4) and highest in Ireland (86.3%, 95% CI 84.9–87.7). Relative survival was significantly lower in England when compared with Belgium (adjusted RER 1.45, 95% CI 1.27–1.66, Table 3, Fig. 2b). For patients with stage III BC, relative survival was lowest in England (48.2%) and highest in Belgium (60.1%). England, Ireland and Greater Poland showed a significantly worse relative survival compared with Belgium (Table 3, Fig. 2c).

**Table 3:** Five-year relative survival and RER stratified by stage

	RS	95% CI	Crude RER	95% CI	*P*	Adjusted RER	95% CI	*P*
Stage I								
Belgium	97.3	96.2–98.1	Reference			Reference		
Greater Poland	103.2	103.2–103.3	NA#		0.543	0.18#	0.001–3000#	0.996
Ireland	99.4	89.0–100.0	NA#		0.702	0.43#	0.001–377#	0.805
The Netherlands	96.0	95.5–96.5	0.81	0.24–2.72	0.402	1.32	0.46–3.84	0.547
England	93.4	93.1–93.7	1.13	1.04–6.10	0.004	2.96	1.30–6.72	< 0.001
Stage II								
Belgium	85.2	84.3–86.1	Reference			Reference		
Ireland	86.3	84.9–87.7	0.92	0.69–1.23	0.574	0.94	0.72–1.22	0.625
The Netherlands	82.5	82.0–83.1	1.10	0.94–1.30	0.224	1.02	0.87–1.18	0.828
Greater Poland	85.3	80.7–88.9	1.26	0.72–2.20	0.418	1.46	0.79–2.69	0.227
England	79.1	78.8–79.4	1.43	1.24–1.66	< 0.001	1.45	1.27–1.66	< 0.001
Stage III								
Belgium	60.1	58.7–61.7	Reference			Reference		
Greater Poland	58.5	52.7–63.8	1.33	0.91–1.95	0.139	1.51	1.05–2.18	0.026
The Netherlands	55.1	54.1–56.0	1.24	1.00–1.52	0.046	1.07	0.90–1.27	0.418
Ireland	53.5	51.3–55.7	1.40	1.18–1.67	< 0.001	1.27	1.07–1.50	0.007
England	48.2	47.8–48.7	1.56	1.40–1.74	< 0.001	1.46	1.31–1.62	< 0.001

Countries were ranked according to the sum of proportions of each given treatment and the country with the highest sum of given treatment was assigned as reference country. n/N: numbers of events/numbers at risk, RS: 5-year relative survival, 95% CI: 95% confidence interval, crude RER: univariate relative excess risk, adjusted RER: multivariable relative excess risk, adjusted for the following confounders: age (continuous), year of diagnosis, grade, morphology. NA: not addressed. # Owing to low excess mortality, RER could not be interpreted

### Treatment patterns and survival differences

As shown in Fig. 2a, representing stage I BC, the proportion of patients receiving adjuvant endocrine therapy was considerably lower in the Netherlands, whereas all other treatment modalities were comparable. No corresponding differences in adjusted RERs were observed. For stage II BC, no evident pattern between treatment and survival outcomes between countries was observed. For stage III BC, the proportion of patients receiving chemotherapy was substantially lower in the Netherlands compared with Belgium, whereas other treatment modalities did not differ greatly. Relative survival was not significantly different between Belgium and the Netherlands (Fig. 2c). However, the proportion of patients receiving any type of surgery was lower in Ireland and England compared with Belgium, whereas other treatment modalities were similar. Concordantly, relative survival was significantly lower in England and Ireland, compared with Belgium.

### Sensitivity analyses

The additional sensitivity analysis showed little variation in treatment outcomes between patients diagnosed in 2008 or 2009 and the complete cohort within a country (Supplementary tables S4 to S6). Supplementary Table S7 shows 5-year relative survival outcomes for all patients diagnosed in 2008 and 2009. The estimated relative survival and the crude and adjusted RERs in this cohort were comparable to estimates found in the complete cohort.

## Discussion

To our knowledge, this is the largest and most recent European population-based study presenting information on stage, tumour characteristics, treatment and survival outcomes in older patients with BC. First, this study showed substantial variation in Europe for treatment of older patients with non-metastatic BC diagnosed between 2000 and 2013. Second, this study reports substantial variation, most pronounced in advanced stage BC, in survival among older patients between European countries. Third, substantially lower proportions of endocrine therapy in patients with stage I BC reported in the Netherlands was not accompanied by poorer survival outcomes; but for stage III BC, poorer survival outcomes were observed in those countries were breast surgery was more frequently omitted. In general, this study suggests poor consensus in the international community on how to optimally treat older patients with breast cancer.

The major strength of our study is that we have the largest available and most detailed population-based data set in Europe. Although a randomised controlled trial (RCT) remains the golden standard for assessment of effectiveness of therapy, real world data has some advantages over RCTs, especially for older patients. It provides a broader and more faithful presentation of patterns of care and comparative effectiveness than RCTs. It furthermore shows a more balanced outcome of benefits and harms of treatment as relative survival represents all excess mortality due to BC: both death directly related to BC itself and death indirectly related to BC.

Limitations in this study should be addressed. Most importantly, data provided by the CRs was not complete for all cases. We performed multiple imputation for missing patient and tumour characteristics. Simulation studies have shown that handling missing data by multiple imputation produces more accurate estimates of relative survival rates, especially for late-stage and high-grade tumours when compared with complete-case analysis^17,18^. Owing to the high proportion of unknown hormone receptor status in England (71.3%), the imputed proportions of hormone receptor status as described in Table 1 might be more uncertain. For Belgium, hormone receptor expression was not available for the cohort at time of analysis but an additional analysis for the year of 2008 showed that hormone receptor distribution was comparable to other countries (data available on request). Moreover, in patients with very high age there might have been poorer diagnostic work-up leading to higher data incompleteness. Although age itself was available for all patients and included as a predictive factor in the multiple imputation, the imputed data may be more uncertain in the oldest patients compared to the younger patients. In England, data on systemic treatment was not complete but completeness improved over time. Owing to incompleteness, non-registered treatment could not be interpreted as not given and therefore this was marked as unknown in tables and figures. For surgical outcomes in England, audits of selected data have shown good completeness but an element of uncertainty should be borne in mind. Another potential weakness is the broad timeline for inclusion of patients and changes in diagnostic procedures and treatment in this period that could have affected variation in survival outcomes. For this reason we performed a sensitivity analyses, but survival rates in the cohort of the years 2008 and 2009 were comparable to complete cohort outcomes. This is in line with previous studies, showing no or limited improvement in survival rates for older patients with BC over the last decade^19–21^. Data on individual factors that could affect treatment outcomes and survival such as comorbidities, patient preferences, and BC subtypes as well as anti-Her2Neu therapy were not available or not complete in the CRs. In addition, there was great variation in the numbers of patients included between the participating countries. This has resulted in less precise estimates for the smallest groups of patients included hampering the interpretation of the data.

The design of this study allowed us to explore possible associations between treatment patterns and survival outcomes. Across Europe, large treatment variation exists and these variations can be used as a natural experiment as variation in assignment to a specific type of treatment was based on country of residence and was therefore not related to the outcome. Tchis enabled us to draw a comparison between treatment patterns and outcomes in an observational setting^8^.

A notable finding was the low proportion of patients receiving adjuvant endocrine therapy with stage I BC in the Netherlands compared with the other countries (19.6% vs. up to 84.6% in Belgium), whereas other treatments did not differ substantially between countries (Fig. 2a). In the Netherlands, endocrine therapy is only recommended in hormone receptor-positive patients with lymph node positive disease or otherwise unfavourable tumour characteristics (high grade or size ≥ 2 cm)^22^, whereas in all the other countries adjuvant endocrine therapy is prescribed in all patients with hormone receptor-positive BC (for an overview of guidelines we refer to Supplementary Table S8). This variation in endocrine therapy was not linked with variation in survival between countries (Belgium 98.6%, Ireland 100.0%, The Netherlands 98.7%), potentially suggesting that adjuvant endocrine therapy does not influence BC-related mortality in a low risk group (Table 3). A previous study comparing Ireland and The Netherlands found similar results^23^. In addition, a population-based study from Denmark identified a subgroup of older patients with low risk BC not treated with adjuvant endocrine therapy that was not at increased risk of mortality^24^. The pattern described in this study potentially suggests that adjuvant endocrine therapy might not contribute to additional survival benefit but further studies are necessary to validate these findings.

In patients with stage III BC, variation in local treatment as well as systemic treatment was apparent. In Belgium, proportions of given local and systemic treatment were high compared with other countries. The proportion of patients in whom breast surgery was omitted was considerably lower in Belgium (22.0%) compared with Ireland (50.8%), whereas other treatment modalities were similar. Only limited evidence is available for the effectiveness of primary endocrine therapy. A meta-analysis showed inferior disease control for 2–3 years after diagnosis but no differences in overall survival compared with surgical treatment followed by adjuvant endocrine therapy^25^. The SIOG guideline recommends that it should only be considered in patients with a life expectancy of < 5 years^2^. In our study, Ireland had a significantly lower survival rate from stage III compared with Belgium (53.5% versus 60.1%, adjusted RER 1.27, 95% CI 1.07–1.50, *P* 0.007). Part of these differences might be explained by variation in breast surgery. It suggests that in this group of high-risk patients breast surgery could result in additional BC survival benefit.

This study demonstrates substantial international variation in type of locoregional treatment, whereas the various guidelines apply largely similar recommendations (Supplementary Table S8). Particularly in Poland, patients with early-stage BC were less likely to receive BCS (Table 2). In those patients with stage II BC who received BCS, we found that radiotherapy was considerably lower in Poland than in other countries. For early-stage BC, omission of radiotherapy after BCS may be justified following publication of the PRIME II trial showing no overall survival difference and a small increase in local recurrences in patients aged 70 years or older with low risk hormone receptor-positive BC^26^. However, no such evidence is available for patients with higher stage disease.

The Netherlands was most conservative in the administration of chemotherapy. For stage III BC, only 10.3% of the Dutch patients received chemotherapy, compared with 35.2% in Belgium. Other international observational studies have found similar patterns^23,27^. The conservative prescription of chemotherapy in the Netherlands can partly be explained by their national guidelines. It states explicitly that patients aged over 70 years should not receive chemotherapy, unless they are considered very fit^22^. No other national or European guidelines use this explicit age criterion^28^ (Supplementary Table S8). The SIOG opposes guidelines using age as a criterion for any treatment as they state that ‘age alone should not dictate any aspect of management of older individuals with BC^2^. Unfortunately, evidence for the effectiveness of chemotherapy in older patients is scarce. In the Early Breast Cancer Trialists’ Collaborative Group (EBCTCG) polychemotherapy overview patients aged 70 years or older were significantly underrepresented. Despite this, the EBCTCG did not find evidence for differences in the effectiveness of chemotherapy for (fit) older patients^29^. Two clinical trials assessing the effect of chemotherapy versus no chemotherapy in older patients with BC were closed prematurely due to poor accrual^30,31^. This also demonstrates the difficulty of performing trials in older patients. Although this study showed that relative survival was lower in The Netherlands (55.1%) compared with Belgium (60.1%), this difference was not significant after adjusting for confounders. Whether chemotherapy could be beneficial in a broader selection of older patients and if it should be offered more frequently in countries with low proportions of chemotherapy remains debatable.

In addition to given treatment, other factors could explain variation in both treatment and relative survival between countries. These include access to and quality of health care, variation in general health and comorbidities and variation in BC subtypes between countries. For instance, national wealth and total national expenditure on health are related to BC guideline adherence and BC survival^27^. In Poland, the St. Gallen Consensus Conference guidelines were used during 2008 and 2009 but adherence to guidelines was affected by suboptimal reimbursement of treatment costs^32^. This could explain poorer survival outcomes for Greater Poland. The EUROCARE-5 study attributed lower survival outcomes in the UK partly to poor access to health care and hence a higher proportion of advanced stage of disease^19^. However, when looking further within specific stages, variation in survival was still apparent in our study. Furthermore, it has been suggested that cancer survival correlates with general health and burden of comorbidities^33^. For instance, if patients are unfit for surgery, radiotherapy or chemotherapy due to comorbidities unrelated to cancer itself, it can also affect cancer-related outcomes. Unfortunately, CRs could not provide us with comprehensive or comparable information on comorbidities for individual patients. Further information on other factors such as comorbidities and quality of life, may be key to gaining a better understanding of treatment processes and patient related outcomes. Additional studies should address the relationship between geriatric characteristics, comorbidities, cancer treatment and quality of life and survival outcomes to bridge the knowledge gap for a rapidly growing older population where more evidence-based treatment is urgently needed. Moreover, cultural factors across countries both in patient preferences and health care professionals could impact decision making in cancer treatment. For instance, we hypothesise that primary endocrine therapy is more common in the United Kingdom and Ireland because trials investigating this treatment have mostly been performed in these countries and this might have enhanced the enthusiasm to propose this type of treatment by health care providers^25^. Moreover, patient preferences for treatment might vary between younger and older patients and there might be differences in these preferences across countries. For the majority of older patients maintaining or increasing quality of life becomes more important than increasing length of life^34^. The burden of frequent hospital visits associated with radiotherapy and the risk of a second surgery are treatment-related aspects that withhold some older patients to undergo BCS^35^. Although a majority of patients would accept adjuvant chemotherapy, older patients are less willing to trade of cognitive or physical capacity for survival benefit^36,37^.

With this study from the EURECCA BC group, we showed large variation in the treatment of older patients with BC between European countries. This implies a lack of consensus in the international community on how to optimally treat older patients with BC, reflecting the lack of evidence-based knowledge and the struggle in clinical practice to treat the very heterogeneous older population. Overall, this study shows that for older patients with low-risk BC, differences in adjuvant endocrine therapy do not appear to impact survival outcomes potentially, suggesting overtreatment of these low risk patients with adjuvant endocrine therapy. On the other hand, variation in the omission of breast surgery in older patients with high risk BC appeared to impact survival substantially, indicating potential undertreatment in this high risk group. Balancing risk of death owing to BC and risk of death owing to other causes seems essential for personalised treatment of older patients with BC.

## Electronic supplementary material

Supplementary material

## Availability of data

Patient level data are available upon request from the separate participating cancer registries. Access to the full data set will be provided if each participating cancer registry gives permission for data sharing. Statistical code is available upon request with the corresponding author.
