# Supplementary material for: Variation in treatment and survival of older patients with non-metastatic breast cancer in five European countries: a population-based cohort study from the EURECCA Breast Cancer Group
Source: Br J Cancer. 2018 Jun 7;119(1):121–9. doi: 10.1038/s41416-018-0090-1 (PMC6035184; doi:10.1038/s41416-018-0090-1)
Supplement: Supplementary file 1 — Supplementary material [file 41416_2018_90_MOESM1_ESM.docx]

**Supplementary Table S1. Coverage, time period and methods of data collection and follow up for CRs**

| **Country or region** | **Name of data registry** | **Incidence years** | **Approximate coverage of country or region (%)** | **Consecutive data** | **Identification of breast cancer diagnosis** | **Collection of data on treatment** | **Collection of data on vital status** | **Date of**  **last follow**  **up** |
| --- | --- | --- | --- | --- | --- | --- | --- | --- |
| The Netherlands | The Netherlands Cancer Registry | 2000-2010 | 100% | Yes | Linkage with Dutch histopathology and cytopathology registry and national hospital discharge databank | Abstraction from individual patient records by the cancer registry | Linkage with municipal registry | 1-1-2015 |
| Belgium | Belgian Cancer Registry | 2007-2010 | 100% (estimated case completeness: 98%) | Yes | Linkage with data of oncological care programs and laboratories for pathological anatomy | Linkage on patient level with health insurance databases (medical claims data) | Linkage with Crossroads Bank for Social Security | 1-7-2015 |
| Ireland | National Cancer Registry Ireland | 2003-2009 | 100% nominal (98% case completeness) | Yes | Pathology reports & patient records from all hospitals & other treatment centres (active & passive registration); some additional cases from death certificates | Abstraction from patient notes & pathology reports by the cancer registry, additional linkage to prescription data to improve completeness of hormonal therapy data | Linkage with national death certificate database | 31-12-2015 |
| England | Public Health England | 2000-2013 | 98% | Yes | All treating and diagnosing institutions send details of registered tumours to PHEs National Cancer Registration Service, which are cross-referenced to each other and national databases. | The treatment records for each tumour are added to by the National Cancer Registry Service. | Linkage with the Office for National Statistics. | 31-12-2015 |
| Greater Poland | Greater Poland Cancer Registry | 2008-2009 | 100% | Yes | Information from cancer case report card, supplementary information from Greater Poland Cancer Centre patient records and Poznań University of Medical Sciences Pathology Department | Information from cancer case report card, supplementary information from Greater Poland Cancer Centre patient records. | Linkage with municipal registry - Central Statistical Office of Poland. | 31-12-2013 |

|  | Axillary surgery |  | Radiotherapy | |  | Endocrine therapy | |  | Chemotherapy | |
| --- | --- | --- | --- | --- | --- | --- | --- | --- | --- | --- |
|  | Yes % |  | Yes % | Unknown % |  | Yes % | Unknown % |  | Yes % | Unknown % |
| Stage I |  |  |  |  |  |  |  |  |  |  |
| The Netherlands | 89.7 |  | 92.4 | 0.0 |  | 19.6 | 0.0 |  | 0.5 | 0.0 |
| Belgium | 94.3 |  | 87.7 | 0.0 |  | 84.6 | 0.0 |  | 5.4 | 0.0 |
| Ireland | 84.4 |  | 74.7 | 0.0 |  | 79.5 | 0.0 |  | 6.0 | 0.0 |
| England | 89.2 |  | 44.6 | 55.4 |  | 47.5 | 52.5 |  | 5.9 | 94.1 |
| Greater Poland | 85.9 |  | 75.3 | 0.0 |  | 68.9 | 0.0 |  | 11.4 | 0.0 |
| Stage II |  |  |  |  |  |  |  |  |  |  |
| The Netherlands | 92.8 |  | 88.8 | 0.0 |  | 64.5 | 0.0 |  | 2.2 | 0.0 |
| Belgium | 94.3 |  | 84.8 | 0.0 |  | 79.5 | 0.0 |  | 16.1 | 0.0 |
| Ireland | 91.6 |  | 77.4 | 0.0 |  | 75.3 | 0.0 |  | 19.4 | 0.0 |
| England | 92.8 |  | 48.9 | 51.1 |  | 47.9 | 52.1 |  | 14.0 | 86.0 |
| Greater Poland | 87.8 |  | 56.5 | 0.0 |  | 65.4 | 0.0 |  | 23.1 | 0.0 |
| Stage III |  |  |  |  |  |  |  |  |  |  |
| The Netherlands | 94.2 |  | 85.4 | 0.0 |  | 70.7 | 0.0 |  | 10.3 | 0.0 |
| Belgium | 95.3 |  | 81.2 | 0.0 |  | 74.0 | 0.0 |  | 35.2 | 0.0 |
| Ireland | 87.9 |  | 73.2 | 0.0 |  | 72.6 | 0.0 |  | 31.6 | 0.0 |
| England | 92.5 |  | 38.0 | 62.0 |  | 43.8 | 56.2 |  | 28.1 | 71.9 |
| Greater Poland | 94.0 |  | 100.0 | 0.0 |  | 62.6 | 0.0 |  | 42.7 | 0.0 |

**Supplementary Table S2. Proportional distribution of locoregional and systemic treatment modalities by stage of disease**

^Axillary surgery: % of patients receiving axillary surgery if they received any type of breast surgery; radiotherapy: % of patients receiving radiotherapy if they have received breast conserving surgery; endocrine therapy: % of patients receiving endocrine therapy if they have received any type of breast surgery; chemotherapy: % of patients receiving chemotherapy if they have received any type of breast surgery.^

|  | Any type of breast surgery | Primary endocrine therapy | No treatment | Unknown |
| --- | --- | --- | --- | --- |
|  | % | % | % | % |
| Stage I |  |  |  |  |
| The Netherlands | 88.3 | 9.4 | 2.3 | 0.0 |
| Belgium | 88.9 | 8.0 | 3.1 | 0.0 |
| Ireland | 82.2 | 15.1 | 2.7 | 0.0 |
| England | 75.8 | 15.6 | 0.0 | 8.6 |
| Greater Poland | 97.5 | 2.0 | 0.0 | 0.4 |
| Stage II |  |  |  |  |
| The Netherlands | 81.8 | 15.6 | 2.6 | 0.0 |
| Belgium | 83.1 | 12.6 | 4.3 | 0.0 |
| Ireland | 78.8 | 17.9 | 3.3 | 0.0 |
| England | 71.9 | 18.3 | 0.0 | 9.9 |
| Greater Poland | 91.1 | 6.5 | 2.0 | 0.4 |
| Stage III |  |  |  |  |
| The Netherlands | 69.9 | 23.6 | 6.5 | 0.0 |
| Belgium | 78.0 | 15.1 | 6.9 | 0.0 |
| Ireland | 49.2 | 39.2 | 11.6 | 0.0 |
| England | 55.9 | 24.9 | 0.0 | 19.3 |
| Greater Poland | 95.4 | 1.8 | 1.8 | 1.0 |

**Supplementary Table S3. Proportional distribution of patients receiving breast surgery, primary endocrine therapy or no therapy by stage of disease**

**Supplementary Table S4. Proportional distribution of most extensive breast surgery for patients diagnosed in 2008 or 2009 by stage of disease**

|  | No surgery | BCS | Mastectomy | Not specified |
| --- | --- | --- | --- | --- |
|  | % | % | % | % |
| Stage I |  |  |  |  |
| The Netherlands | 13.3 | 51.9 | 34.9 | 0.0 |
| Belgium | 11.5 | 65.1 | 23.3 | 0.0 |
| Ireland | 24.3 | 56.8 | 18.9 | 0.0 |
| England | 24.2 | 50.3 | 25.5 | 0.0 |
| Greater Poland | 2.5 | 21.1 | 52.4 | 24.0 |
| Stage II |  |  |  |  |
| The Netherlands | 22.4 | 22.5 | 55.2 | 0.0 |
| Belgium | 16.5 | 36.6 | 46.9 | 0.0 |
| Ireland | 26.3 | 34.0 | 39.7 | 0.0 |
| England | 28.9 | 27.0 | 44.1 | 0.0 |
| Greater Poland | 8.9 | 8.3 | 66.1 | 16.7 |
| Stage III |  |  |  |  |
| The Netherlands | 28.1 | 10.0 | 62.0 | 0.0 |
| Belgium | 23.1 | 14.6 | 62.3 | 0.0 |
| Ireland | 53.4 | 8.0 | 38.7 | 0.0 |
| England | 42.0 | 10.5 | 47.5 | 0.0 |
| Greater Poland | 4.6 | 3.4 | 81.8 | 10.2 |

^BCS= breast conserving surgery^

|  | Axillary surgery |  | Radiotherapy | |  | Endocrine therapy | |  | Chemotherapy | |
| --- | --- | --- | --- | --- | --- | --- | --- | --- | --- | --- |
|  | Yes % |  | Yes % | Unknown % |  | Yes % | Unknown % |  | Yes % | Unknown % |
| Stage I |  |  |  |  |  |  |  |  |  |  |
| The Netherlands | 96.2 |  | 93.2 | 0.0 |  | 29.8 | 0.0 |  | 1.1 |  |
| Belgium | 94.4 |  | 88.6 | 0.0 |  | 84.5 | 0.0 |  | 5.4 | 0.0 |
| Ireland | 90.5 |  | 81.9 | 0.0 |  | 77.5 | 0.0 |  | 6.9 | 0.0 |
| England | 91.6 |  | 38.4 | 61.6 |  | 46.1 | 53.9 |  | 6.7 | 0.0 |
| Greater Poland | 85.9 |  | 75.3 | 0.0 |  | 68.9 | 0.0 |  | 11.4 | 93.3 |
| Stage II |  |  |  |  |  |  |  |  |  | 0.0 |
| The Netherlands | 95.6 |  | 89.9 | 0.0 |  | 72.4 | 0.0 |  | 2.8 |  |
| Belgium | 93.7 |  | 84.9 | 0.0 |  | 79.6 | 0.0 |  | 17.0 | 0.0 |
| Ireland | 94.2 |  | 85.2 | 0.0 |  | 75.4 | 0.0 |  | 23.4 | 0.0 |
| England | 94.4 |  | 44.0 | 56.0 |  | 45.6 | 54.4 |  | 15.6 | 0.0 |
| Greater Poland | 87.8 |  | 56.5 | 0.0 |  | 65.4 | 0.0 |  | 23.1 | 84.4 |
| Stage III |  |  |  |  |  |  |  |  |  | 0.0 |
| The Netherlands | 96.6 |  | 89.9 | 0.0 |  | 70.5 | 0.0 |  | 16.0 |  |
| Belgium | 95.4 |  | 79.7 | 0.0 |  | 73.1 | 0.0 |  | 37.0 | 0.0 |
| Ireland | 94.6 |  | 85.5 | 0.0 |  | 71.0 | 0.0 |  | 37.1 | 0.0 |
| England | 94.3 |  | 33.1 | 66.9 |  | 40.1 | 59.9 |  | 29.7 | 0.0 |
| Greater Poland | 94.0 |  | 100.0 | 0.0 |  | 62.6 | 0.0 |  | 42.7 | 70.3 |

**Supplementary Table S5. Proportional distribution of locoregional and systemic treatment modalities for patients diagnosed in 2008 or 2009**

^Axillary surgery: % of patients receiving axillary surgery if they received any type of breast surgery; radiotherapy: % of patients receiving radiotherapy if they have received breast conserving surgery; endocrine therapy: % of patients receiving endocrine therapy if they have received any type of breast surgery; chemotherapy: % of patients receiving chemotherapy if they have received any type of breast surgery.^

**Supplementary Table S6. Proportion of patients diagnosed in 2008 and 2009 receiving breast surgery, primary endocrine therapy or no therapy by stage of disease**

|  | Any type of breast surgery | Primary endocrine therapy | No treatment | Unknown |
| --- | --- | --- | --- | --- |
|  | % | % | % | % |
| Stage I |  |  |  |  |
| The Netherlands | 86.7 | 11.5 | 1.8 | 0.0 |
| Belgium | 88.5 | 8.6 | 2.9 | 0.0 |
| Ireland | 75.7 | 20.6 | 3.7 | 0.0 |
| England | 75.8 | 15.8 | 0.0 | 8.4 |
| Greater Poland | 97.5 | 2.0 | 0.0 | 0.4 |
| Stage II |  |  |  |  |
| The Netherlands | 77.6 | 19.5 | 2.8 | 0.0 |
| Belgium | 83.5 | 12.4 | 4.1 | 0.0 |
| Ireland | 73.7 | 23.7 | 2.5 | 0.0 |
| England | 71.1 | 18.5 | 0.0 | 10.4 |
| Greater Poland | 91.1 | 6.5 | 2.0 | 0.4 |
| Stage III |  |  |  |  |
| The Netherlands | 71.9 | 22.4 | 5.7 | 0.0 |
| Belgium | 76.9 | 16.4 | 6.6 | 0.0 |
| Ireland | 46.6 | 43.3 | 10.1 | 0.0 |
| England | 58.0 | 22.4 | 0.0 | 19.6 |
| Greater Poland | 95.4 | 1.8 | 1.8 | 1.0 |

|  |  |  | RS | 95% CI |  | Crude RER | 95% CI | *P* |  | Adjusted RER | 95% CI | *P* |
| --- | --- | --- | --- | --- | --- | --- | --- | --- | --- | --- | --- | --- |
| Stage I | |  |  |  |  |  |  |  |  |  |  |  |
|  | Belgium |  | 97.9 | 96.1-98.9 |  | reference |  |  |  | reference |  |  |
|  | Greater Poland |  | 103.2 | 103.2-103.3 |  | NA# |  | 0.998 |  | 1.71# | 0.02-117 | 0.801 |
|  | Ireland |  | 100.6 | 100.6-100.6 |  | 0.92# | 0.11-7.48 | 0.938 |  | 0.28# | 0.001-1600# | 0.926 |
|  | The Netherlands |  | 95.9 | 94.6-96.8 |  | 0.61 | 0.21-1.81 | 0.374 |  | 1.41 | 0.35-5.61 | 0.617 |
|  | England |  | 94.0 | 93.2-94.7 |  | 0.99 | 0.41-2.40 | 0.981 |  | 2.42 | 0.54-10.93 | 0.229 |
| Stage II | |  |  |  |  |  |  |  |  |  |  |  |
|  | Belgium |  | 85.1 | 83.8-86.4 |  | reference |  |  |  | reference |  |  |
|  | Ireland |  | 86.8 | 84.2-89.0 |  | 0.70 | 0.41-1.20 | 0.193 |  | 0.84 | 0.53-1.33 | 0.456 |
|  | The Netherlands |  | 81.4 | 80.1-82.7 |  | 1.08 | 0.84-1.38 | 0.559 |  | 1.11 | 0.88-1.40 | 0.401 |
|  | Greater Poland |  | 85.3 | 80.7-88.9 |  | 1.19 | 0.67-2.11 | 0.548 |  | 1.40 | 0.73-2.66 | 0.308 |
|  | England |  | 80.5 | 79.8-81.1 |  | 1.20 | 0.98-1.47 | 0.080 |  | 1.33 | 1.10-1.61 | 0.004 |
| Stage III | |  |  |  |  |  |  |  |  |  |  |  |
|  | Belgium |  | 60.9 | 58.7-62.9 |  | reference |  |  |  | reference |  |  |
|  | Greater Poland |  | 58.5 | 52.7-63.8 |  | 1.37 | 0.93-2.02 | 0.113 |  | 1.59 | 1.38-1.79 | 0.015 |
|  | The Netherlands |  | 57.1 | 54.9-59.2 |  | 1.11 | 0.85-1.45 | 0.442 |  | 1.12 | 0.87-1.44 | 0.355 |
|  | Ireland |  | 54.7 | 50.7-58.6 |  | 1.35 | 1.00-1.83 | 0.053 |  | 1.36 | 1.02-1.82 | 0.038 |
|  | England |  | 50.6 | 49.3-51.8 |  | 1.50 | 1.26-1.78 | <0.001 |  | 1.50 | 1.27-1.76 | <0.001 |

**Supplementary Table S7. Five year relative survival for patients diagnosed in 2008 and 2009**

^Countries were ranked according to the sum of proportions of each given treatment and the country with the highest sum of given treatment was assigned as reference country. n/N: numbers of events/numbers at risk, RS: five-year relative survival, 95% CI: 95% Confidence Interval, crude RER: univariate relative excess risk, adjusted RER: multivariable relative excess risk, adjusted for the following confounders: age (continuous), year of diagnosis, grade, morphology. NA: not addressed. # Due to low excess mortality, RER could not be interpreted.^

|  | **The Netherlands**  (1, 2) | **Ireland**  (3) | **Belgium** (4) | **England**  (5, 6) | **Poland**  (7, 8) |
| --- | --- | --- | --- | --- | --- |
| **Local surgery** | BCS: in all patients with T1-2; N0-1 breast cancer | BCS: unifocal, anticipated acceptable cosmetic result, not occurring in 1st/2nd trimester of pregnancy, possibility to obtain histologically clear margins (5mm), patient preference, no contraindications to RT | BCS stage I or II | BCS: tumours <2.5 cm | 2007: No BCS for multifocal disease Exclusion of multifocal disease by MRI may improve  selection for conservative surgery. |
|  | Mastectomy: patient preference  contraindications to BCS and/or RT | Mastectomy if contraindications to BCS and/or RT |  |  |  |
| **Axillary surgery** | Sentinel node in all patients, except when axillary metastasis is proven, or with a ≥ T2 and multicentric primary tumor, or the patient had previous axillary surgery | 2000: Sentinel node still investigational, ALND (level I and II or I-III) in all patients  2007:  - sonographic normal LN's and cytology not performed/negative: SN  - proved LN involvement (by cytology or SN): axillary clearance (levels I and II or I-III) | Sentinel node: T < 3 cm, clinical and sonographic normal  lymfenodes  ALND when:  Positive sentinel node  Large T2 (3cm), or T3-4 Inflammatory BC  Clinical palpable lymph nodes  Multipel tumors | 1996: Sentinel node biopsy is an accurate diagnostic tool  in patients with clinically node negative breast  cancer  ALND in:  Positive SLN and clinical proven positive lymph nodes | 2007: Sentinel node biopsy was accepted as reliable and safe even in elderly patients  ALND in:  Positive sentinel node (including micrometastasis) and clinical proven or palpable positive lymph nodes |
|  | Axillary lymph node dissection after positive sentinel node or in case of contraindications to sentinel node | 2000: sentinel node still investigational, ALND (level I and II or I-III) in all patients  2007:  - sonographic normal lymph nodes and cytology not performed/negative: sentinel node  - proved sentinel node involvement (by cytology or sentinel lymph node biopsy): axillary clearance (levels I and II or I-III)  After BCS |  | 2009:  ALND if:  the sentinel node is positive (macrometastasis or micrometastasis), further axillary treatment (axillary dissection  or radiotherapy) as well as adjuvant systemic therapy is recommended |  |
| **Radiation therapy** | Always after BCS  After mastectomy: irradical resection, cT4, pT3 and ≥ pN2, involvement of pectoral muscle, or a positive axillary apex | After BCS  After mastectomy in cases of high risk of local chest wall recurrence (tumor ≤3 mm of pectoral fascia or >4 positive axillary nodes) | After BCS  After Mastectomy: when high risk for local recurrence:  4 or more positive lymph nodes  Irradical resection | 1996: After BCS, unless radiotherapy  is contra-indicated or the patient is entered into a  clinical trial. After mastectomy: consider radiotherapy | 2007: Radiation therapy is clearly indicated after breast conserving surgery  including a boost in younger patients.  post-mastectomy radiation therapy for all patients with 4 or more involved  lymph nodes |
|  |  |  |  | 2009: After BCS, unless radiotherapy  is contra-indicated or the patient is entered into a  clinical trial. |  |
| **Endocrine therapy** | Postmenopausal N+ ER+/PR+ | N+, ER/PR+ | Postmenopausal ER+/PR+ | 1996: All patients with ER+ disease | 2007: all patients with ER+ disease |
|  | N0, tumour size ≤ 1 cm: none | N0, tumour size ≤ 1 cm, ER/PR+, grade 1: none or tamoxifen |  | 2009: All patients with ER+ invasive breast carcinoma can potentially benefit from endocrine therapy |  |
|  | Since 2008: N0 tumour size 1-2 cm, grade 3  N0 tumour size 2-3 cm, grade 2-3  N0 tumour size ≥ 3 cm. | N0, tumour size ≥ 1 cm, ER/PR+, grade > 1 |  |  |  |
| **Chemotherapy** | Premenopausal N+ | All N0 ER+/PR+ patients except "elderly": tumour size >1cm, all grades (before endocrine therapy) |  | 1998:No specific recommendation: especially in premenopausal women with  ER negative tumours | 2007: Features that raise doubt about  the adequacy of endocrine therapy alone include relatively  lower expression of steroid hormone receptor, involvement  (and particularly extensive involvement) of axillary lymph  nodes, higher grade or proliferative markers, larger tumor  size and extensive peri-tumoral vascular invasion. |

1. NABON. Richtlijn Mammacarcinoom versie 2.0 2012 [updated 2/13/2012. Available from: <www.oncoline.nl/mammacarcinoom>.

2. CBO. Richtlijn Mammacarcinoom 2005 2005.

3. O’Higgins. National Quality Assurance Standards for Symptomatic Breast Disease Services—Developing Quality Care for Breast Services in Ireland. Health Information and Quality Authority; 2007.

4. Gezondheidszorg FKvd. Nationale richtlijn voor de behandeling van borstkanker. 2007.

5. Catterall A. Guidelines for surgeons in the management of symptomatic breast disease in the United Kingdom. European journal of surgical oncology : the journal of the European Society of Surgical Oncology and the British Association of Surgical Oncology. 1996;22(2):202.

6. Surgical guidelines for the management of breast cancer. European journal of surgical oncology : the journal of the European Society of Surgical Oncology and the British Association of Surgical Oncology. 2009;35 Suppl 1:1-22.

7. Goldhirsch A, Wood WC, Gelber RD, Coates AS, Thurlimann B, Senn HJ. Progress and promise: highlights of the international expert consensus on the primary therapy of early breast cancer 2007. Annals of oncology : official journal of the European Society for Medical Oncology / ESMO. 2007;18(7):1133-44.

8. Wysocki KHAAŚAWM. Breast cancer treatment outcomes, therapy options and costs in Poland (2005–2007)2014. 33-9 p.
